# Editorial: Genetic markers identification for animal production and disease resistance

**DOI:** 10.3389/fgene.2023.1243793

**Published:** 2023-07-12

**Authors:** Ibrar Muhammad Khan, Adnan Khan, Hongyu Liu, Muhammad Zahoor Khan

**Affiliations:** ^1^ Anhui Province Key Laboratory of Embryo Development and Reproduction Regulation, Anhui Province Key Laboratory of Environmental Hormone and Reproduction, School of Biological and Food Engineering, Fuyang Normal University, Fuyang, China; ^2^ Genome Analysis Laboratory of the Ministry of Agriculture, Agricultural Genomics Institute at Shenzhen, Chinese Academy of Agricultural Sciences, Shenzhen, China; ^3^ Anhui Provincial Laboratory of Local Livestock and Poultry Genetical Resource Conservation and Breeding, College of Animal Science and Technology, Anhui Agricultural University, Hefei, China; ^4^ Department of Animal Breeding and Genetics, Faculty of Veterinary and Animal Sciences, The University of Agriculture, Dera Ismail Khan, Pakistan

**Keywords:** production traits, genomics, genetic markers, animal health, markers assisted selection

It is well established that several production traits and diseases are polygenetic in nature (van Rheenen et al., 2019). Traditional methods have proven inadequate in effectively enhancing production performance and disease control in animals. Thus, identification of markers and the underlying mechanism for controlling these phenotypes is the main focus of research today in animal science (Goddard and Hayes, 2009). Various genetic approaches including RNA-sequencing (Augustino et al., 2020; Khan et al., 2020; Liu et al., 2022), whole-genome sequencing, mapping of quantitative trait loci (QTL) (Uemoto et al., 2021), candidate gene analysis (Yang et al., 2016) and genome-wide association study (GWAS) (Kai-Yuan et al.) (Wang et al., 2022) have been utilized to identify fundamental genes or their polymorphisms correlated with animal production and disease resistance phenotypic traits in animals (Ma et al., 2021; Khan et al., 2023). The utilization of markers for milk quality, production traits, disease resistance (Khan et al., 2022; Yang et al., 2022), thermo-tolerance, fertility, and carcass quality in cattle plays a crucial role in enhancing their health and productivity (Khan et al., 2021). These markers enable targeted selection and breeding programs, optimizing production and ensuring reduce infection risks in animals.

Li et al. investigated the role of ACSL1 in fatty acid synthesis and metabolism, specifically focusing on its impact on highly unsaturated fatty acids in beef. They transfected bovine preadipocytes with si-ACSL1 and NC-ACSL1, and used RNA-Seq to identify miRNAs associated with unsaturated fatty acid synthesis. By analyzing the miRNA-mRNA interaction network, several key miRNA-mRNA targeting relationships were identified, including novel-m0035-5p—ACSL1, novel-m0035-5p—ELOVL4, miR-9-x—ACSL1, bta-miR-677—ACSL1, miR-129-x—ELOVL4, and bta-miR-485—FADS2. These specific miRNAs have the potential to regulate unsaturated fatty acid synthesis in bovine adipocytes by targeting these specific genes. A study conducted by Du et al. utilized resequencing techniques to identify 21 single nucleotide polymorphisms (SNPs) in the PKLR gene of Chinese Holstein cows. Their analysis demonstrated significant associations between these SNPs and milk yield, fat and protein yields, as well as protein percentage. Additionally, in a separate study by Jia et al., two specific SNPs (SNP g.54362761A>G and g.54326411T>C) in the SEC13 gene were documented. Notably, Jia et al. observed a significant association between these SNPs and milk production phenotypic traits in dairy cattle. Another study by Zhai et al. revealed that intrauterine exosomes in bovine pregnancy play a role in embryo development and implantation. Analysis of miRNA expression in these exosomes revealed significant differences between donor and recipient cows, with 22 miRNAs upregulated and 38 downregulated in the donor group. The identified miRNAs were associated with biological processes and pathways related to embryo implantation and endometrial development, suggesting their potential influence on the uterine microenvironment for successful implantation of embryos.

Luo et al. examined HIAT1 expression in Lanzhou fat-tailed (LFT) sheep and found it widely expressed, with high levels in the testis. They also discovered a 9-bp insertion mutation (rs1089950828) in Luxi black-headed (LXBH) and Guiqian semi-fine wool (GSFW) sheep breeds. The mutation was associated with morphometric traits in LXBH and GSFW sheep. Yearling ewes with a heterozygous genotype (ID) had smaller body sizes, while yearling rams and adult ewes showed better growth performance. Furthermore, the authors suggested the potential use of this mutation for marker-assisted selection in Chinese sheep populations.

Fine wool production significantly contributes to the revenue of extensive wool sheep production in the United States. In their study, Becker et al. focused on Rambouillet sheep, renowned for their high-quality wool, aiming to unravel the genetic basis of wool characteristics. Over a 3-year period, wool samples and DNA were collected from rams participating in performance tests. Genome-wide association studies revealed significant genetic markers linked to wool quality traits, specifically on chromosomes 1, 2, 4, 15, and 19. The study also identified strong correlations between various wool characteristics, such as clean fleece weight and staple length. Moreover, certain wool traits exhibited associations with body weight and scrotal circumference. These findings provide valuable insights for Rambouillet sheep breeders, aiding them in making informed decisions regarding targeted selection and breeding to enhance wool quality.

Climate changes, particularly extreme weather events, can have a negative impact on livestock production. To understand the genetic mechanisms behind sheep prolificacy traits in the Taklimakan Desert, Zhang et al. conducted a study using Pishan Red Sheep (PRS) and Qira Black Sheep (QR). Blood samples were collected from these sheep, and DNA was extracted and analyzed using the Illumina Ovine SNP50 chip. The study revealed the genetic characteristics of PRS, including linkage disequilibrium, effective population size, and genetic markers identified through iHS and FST analyses. A total of 29 genes were found to be common in both analyses. These findings provide valuable insights for the conservation of sheep genetic resources and molecular breeding in desert environments.

Kai-Yuan et al. explored the roles of long noncoding RNAs (lncRNAs) in melanocytes, which are responsible for determining skin and hair color through melanin production. Melanocytes from Boer goat skins were isolated and characterized using various staining and immunohistochemical techniques. A phenotypic analysis revealed significant differences in the behavior and melanin production of melanocytes from goats with white and brown hair. RNA sequencing was performed, leading to the identification of candidate lncRNAs and mRNAs involved in stage-specific melanogenesis. Functional enrichment analysis highlighted the involvement of miRNA precursors and cisregulatory effects of lncRNAs. The study also proposed multiple lncRNA-mRNA networks related to melanocyte migration, proliferation, and melanogenesis, offering new insights into mammalian pigmentation.

A recent experimental trial conducted by Zhang et al. aimed to investigate the genetic diversity and population structure of pigs in Anhui Province, China. The study involved the sequencing of 150 pig genomes from six representative populations, which were then compared to data from Asian wild boars and commercial pigs. The analysis uncovered two distinct ancestral origins of Anhui pigs, namely, the Wannan Spotted pig (WSP) and Wannan Black pig (WBP) from a common ancestor, while the remaining four populations originated from a separate ancestor. The researchers also identified specific genomic regions associated with domestication traits in Anhui pigs, such as reproduction, lipid and meat characteristics, and ear size genes (CABS1, INSL6, MAP3K12, IGF1R, INSR, LIMK2, PATZ1, MAPK1, SNX19, MSTN, MC5R, PRKG1, CREBBP, and ADCY9). These findings not only enhance our understanding of genetic variations among pig populations but also hold promise for future genetic research and the advancement of genome-assisted breeding in pigs and other domesticated animals.

The study conducted by Yao et al. aimed to identify candidate genes associated with mammary gland development in Bactrian camels (*Camelus bactrianus*) through transcriptome analysis. The researchers analyzed the gene expression profiles of the mammary gland tissues and identified differentially expressed genes (DEGs) involved in the development of the mammary gland. Functional enrichment analysis provided insights into the biological processes, molecular functions, and cellular components associated with mammary gland development. Several candidate genes, including those involved in epithelial development, mammary gland morphogenesis, and milk synthesis, were identified. This research contributes to a better understanding of the genetic mechanisms underlying mammary gland development in Bactrian camels, which may have implications for improving camel milk production and reproductive performance.

Kochish et al. investigated the genetic diversity and divergent selection in chickens, focusing on breed-specific patterns related to early myogenesis, nitric oxide metabolism, and post-hatch growth. The researchers analyzed the genetic signatures of various chicken breeds and identified differentially expressed genes (DEGs) associated with these traits. They observed breed-specific patterns in early myogenesis, suggesting unique genetic pathways involved in muscle development across different chicken breeds. Additionally, variations in nitric oxide metabolism and post-hatch growth were found among the breeds. This study sheds light on the genetic basis of these traits in chickens, providing valuable insights for future breeding strategies aimed at enhancing muscle development and growth in poultry. In addition, Ye et al. investigated the association between fat-related traits in chickens and the RGS16 gene. They examined polymorphisms within the RGS16 gene and performed functional validation analysis to gain insights into its role in fat-related traits. The study aimed to understand the potential impact of genetic variations in the RGS16 gene on fat deposition and metabolism in chickens. The findings provide valuable information on the genetic factors influencing fat-related traits in poultry and contribute to a better understanding of the molecular mechanisms underlying fat deposition and metabolism. This research may have implications for improving breeding strategies and the production of leaner and healthier chicken meat.
